# Post-operative pain in molar teeth with symptomatic apical periodontitis using cryotreated neem extract and cryotreated 2.5% sodium hypochlorite: A randomised double-blinded clinical trial

**DOI:** 10.6026/973206300222701

**Published:** 2026-04-30

**Authors:** Ruchi Vashisht, Umesh Kumar, Ashish Kumar Kakkar, Om Parkash Katare, Umesh Kumar Patil, Prabhleen Kaur Brar

**Affiliations:** 1 Department of Conservative & Endodontics, Oral Health Science Centre, PGIMER, Chandigarh, India; 2 Department of Pharmacology, PGIMER, Chandigarh, India; 3 Department of Pharmaceutical Science, Punjab University, Chandigarh, India; 4 Department of Pharmaceutical Science, DR. Harisingh Gour Vishwavidyalaya, Sagar, Madhya Pradesh, India

**Keywords:** Cryotherapy, neem extract, sodium hypochlorite, post-operative pain, root canal irrigation, randomised clinical trial

## Abstract

Post-operative pain following root canal treatment remains a significant clinical concern. Therefore, it is of interest to evaluate the effect of cryotherapy using sodium hypochlorite and neem extract on post-endodontic pain in 108 patients with symptomatic apical periodontitis undergoing single-visit treatment. Participants were divided into four groups: room temperature sodium hypochlorite, cryotreated sodium hypochlorite, room temperature neem extract and cryotreated neem extract and pain were assessed using the Visual Analogue Scale at 24 hours, 48 hours and 7 days. Cryotreated irrigants showed significantly lower pain scores at 24 and 48 hours compared to room temperature groups and neem extract demonstrated comparable efficacy to cryotreated sodium hypochlorite. Thus, the potential of cryotreated neem extract is shown as a biocompatible alternative irrigant for effective post-operative pain reduction.

## Background:

Post-operative pain is a major clinical challenge that follows root canal treatment procedure which usually compromises on patient comfort and acceptance of treatment. It has a multifactorial etiology that is caused by an acute periapical inflammation through mechanical, chemical, or microbial insulation in a procedure or post-anesthetic happens during or following endodontic practices. Its incidence is reported to be between 1.4 to 53 percent and it shows the necessity to provide adequate pain management methods during and after the treatment [1, 2]. However, cryotherapy or the use of low temperatures in the treatment process has been widely employed in both the field of sports medicine and general surgery as a method of treating pain and inflammation. Cryotherapy in the field of dentistry proved promising about post-operative pain management due to periradicular surgeries and endodontic treatment [3, 4- 5]. Different works found improvements represented by reducing the intensity of pain and the level of inflammatory mediators by means of intracanal cryotherapy with cold saline or sodium hypochlorite [6, 7]. Standardization of cryotherapy parameters, including temperature, mode of application and duration, remains an area of ongoing research. In recent years, herbal and natural products have gained increasing attention due to their biocompatibility, anti-inflammatory and antimicrobial properties. Neem (*Azadirachta indica*), commonly known as the margosa tree, contains several bioactive compounds and has been recognized by the US National Academy of Sciences for its potential in addressing global health challenges. Its anti-inflammatory effects and compatibility with human periodontal ligament cells support its potential use as an endodontic irrigant [8, 9]. However, limited evidence exists regarding the effectiveness of cryotreated neem extract in reducing post-operative pain. Therefore, it is of interest to evaluate and compare the effectiveness of vitrified neem extract and vitrified 2.5% sodium hypochlorite in reducing post-operative pain following single-visit root canal treatment.

## Methodology:

## Study design and ethical approval:

This study was conducted as a randomized, double-blind clinical experiment at PGIMER, Chandigarh and was approved by the Institutional Ethics Committee (Approval No: IEC-03/2023-2669). Written informed consent was obtained from all participants prior to their inclusion in the study.

## Study participants:

Individuals aged 18 to 40 years necessitating single-visit root canal therapy for molar teeth with symptomatic apical periodontitis were included. Symptomatic apical periodontitis was identified by moderate to severe pre-operative discomfort (VAS 3-10) and pronounced percussion pain (VAS >6).

## Inclusion criteria:

[1] Patients aged 18 to 40 years who require endodontic therapy and are prepared to offer informed permission.

[2] Molar teeth exhibiting vital pulp identified with symptomatic apical periodontitis.

## Symptomatic apical periodontitis was characterized by:

[1] Pre-operative pain score ranging from 3 to 10 on the Visual Analogue Scale (VAS).

[2] Percussion discomfort level above 6 on the Visual Analog Scale (VAS).

## Exclusion criteria:

[1] Presence of any systemic disease or known allergic reactions.

[2] Current use of analgesics or antibiotic medications.

[3] Pregnant patients.

[4] Retreatment cases.

## Teeth exhibiting any of the following:

[1] Sinus tracts

[2] Open apex

[3] Root resorption

[4] Perforation

[5] Severe periodontal disease (periodontal pockets >3 mm)

[6] Presence of periapical radiolucency

## Computation of sample size:

The required sample size was calculated based on existing literature and guidelines for randomized controlled trials to achieve a study power of 80% (Z1-β=0.84), at a 95% confidence interval (Z1-α/2=1.96), with a precision of 8% (d=0.05) and an assumed pooled standard deviation (σ) of 0.1 (10%). The sample size was determined utilizing the usual procedure for determining sample sizes in comparative studies.

n=(Z_1_-α/2) + Z_1_-B/)^2^)2 σ^2^)/(d)^2^

=(2(1.96+0.84)^2^ x 0.01)/(0.08)^2^

n=0.1568/(0.0064)

n=24.5

Further, by considering an attrition rates (10%), the sample size for each has come out to be approximately 27 participants in each group, which indicates a cumulative sample size of at least 108 participants (for both intervention and control groups, since 24.5*4 =108). Upon receiving clearance from the institutional review board, patients meeting the inclusion criteria were recruited for this study. Pre- operative data for each patient was included in the preformatted patient chart, encompassing age, sex and tooth number prior to treatment. The therapy and research design were elucidated to the eligible patients and informed permission was acquired from the volunteer participants wanting to engage in the study. The sample size was calculated in such a way that it would give an 80 percent power and 95 percent confidence limit regarding a pooled standard deviation of 0.1. Taking into consideration that 10 percent of the participants would drop out, 27 participants were recruited per each group meaning 108 patients in total.

## Blinding and randomization:

Eligible patients will be randomly assigned in a 1:1 ratio to either the active treatment or placebo group using a computer-generated randomization sequence with variable block sizes. Allocation concealment will be guaranteed by use sequentially numbered, sealed, opaque envelopes that carry the group assignment. This investigation will be executed as a double-blind experiment, wherein both participants and result assessors are unaware of the treatment assignment. The mode of assigning patients was random as they were distributed into four groups (n=27). Each group was randomly assigned to groups in a sequence of a computer-generated sequence with variable block sizes.

The sealing of all envelopes was done using SNOSE.

[1] Blinding of the participants: The patients were blinded to the type of irrigant employed.

[2] Evaluator blinding: Group allocation was not known to evaluator who measured post-operative pain.

## Intervention protocol:

All procedures were conducted under rubber dam isolation and local anesthesia. The preparation of the access cavity was succeeded by the cleaning and shape of the root canals. The working length was established with both manual and rotary files.

## Participants were randomly allocated into four groups:

[1] Group A: Conclusive irrigation with 20 ml of 2.5% sodium hypochlorite at ambient temperature, delivered after a 5-minute interval.

[2] Group B: Conclusive irrigation utilizing 20 ml of cryotreated 2.5% sodium hypochlorite (preserved at 2°C-4°C), delivered following a 5-minute interval.

[3] Group C: Concluded irrigation with 20 ml of neem extract at ambient temperature for 5 minutes.

[4] Group D: Conclusive irrigation utilizing 20 ml of cryotreated neem extract (stored at 2°C-4°C), applied for a duration of 5 minutes.

Cryotreated irrigants were stored and maintained at the target temperature using an insulated icebox. Temperature accuracy was ensured using a calibrated thermocouple.

## Preparation of neem extract:

Fresh neem leaves (*Azadirachta indica*) were thoroughly washed, air-dried and then powdered. A total of 300 g of the powdered leaves was macerated in a hydroalcoholic solvent (ethanol: water in a 1:5 v/v ratio) for 24 hours. The mixture was then filtered and the filtrate was concentrated at 50°C under reduced pressure. The final extract was stored in amber-colored containers at room temperature until use.

## Outcome assessment:

Post-operative pain was assessed utilizing the Visual Analogue Scale (VAS) after 24 hours, 48 hours and 7 days post-procedure. Patients were instructed to document the quantity of analgesic pills ingested during the follow-up duration.

## Statistical examination:

The analysis of data was conducted with SPSS software. Continuous variables were represented as mean ± standard deviation. Intergroup comparisons were conducted utilizing analysis of variance (ANOVA) accompanied by post-hoc testing. A p-value below 0.05 was deemed statistically significant.

## Results:

A total of 108 patients aged 18-40 years (mean age: 28.1 ± 4.7 years) were recruited and equally allocated into four groups (n = 27 each). Baseline characteristics, including age, sex distribution and pre-operative VAS scores, showed no statistically significant differences among the groups (p > 0.05) (Table 1 - see PDF). At 24 hours post-treatment, Groups B (cryotreated sodium hypochlorite) and D (cryotreated neem extract) demonstrated significantly lower mean VAS scores (2.1 ± 0.5 and 2.0 ± 0.6, respectively) compared to Groups A and C (4.5 ± 0.6 and 4.3 ± 0.7, respectively) (p < 0.001). A similar trend was observed at 48 hours, with Groups B and D showing lower pain scores (1.2 ± 0.4 and 1.1 ± 0.5, respectively) than Groups A and C (2.8 ± 0.7 and 2.6 ± 0.8, respectively) (p < 0.001). By day 7, pain levels had reduced across all groups with no statistically significant difference (p = 0.08) (Table 1, Figure 1 - see PDF). Analgesic consumption within the first 48 hours was significantly lower in Groups B and D (1.1 ± 0.4 and 1.0 ± 0.3, respectively) compared to Groups A and C (2.4 ± 0.8 and 2.2 ± 0.7, respectively) (p < 0.001). Most participants in Groups B and D required no or only one analgesic tablet, whereas higher consumption (≥2 tablets) was more frequently observed in Groups A and C (Table 1, Figure 2 - see PDF).

## Discussion:

Post-operative pain is a well-documented complication of endodontic therapy, with a reported incidence ranging from 1.4% to 53% despite adherence to standard protocols [1]. It results from acute periapical inflammation triggered by mechanical, chemical, or microbial insults during and after root canal procedures [2]. Effective strategies to minimize this discomfort remain an active area of research in endodontics. Cryotherapy has emerged as a promising adjunct in pain management due to its ability to induce vasoconstriction, reduce nerve conduction velocity and suppress inflammatory mediator release [3, 4]. Several studies have demonstrated its efficacy in reducing post-endodontic pain [5]. Hamza *et al.* [9] showed that intraoral cryotherapy significantly decreased post-operative pain and substance P levels in patients with symptomatic apical periodontitis. Similarly, other clinical trials have reported a significant reduction in pain following the use of intracanal cryotherapy [10, 11, 12- 13]. Our findings are consistent with those of Keskin *et al.* [14], who reported that intracanal cryotherapy reduced inflammatory cytokine and proteolytic enzyme levels. Almasoud *et al.* [15] further suggested that combining cryotherapy with occlusal reduction enhances pain control, highlighting the potential synergistic effects of adjunctive interventions. In parallel, increasing interest has been directed toward biocompatible irrigants such as neem (*Azadirachta indica*) due to their antimicrobial and anti-inflammatory properties [6, 7]. Hosny *et al.* [10] demonstrated that neem was comparable to sodium hypochlorite in reducing postoperative pain and endotoxin levels, while Palwankar *et al.* [11] supported the antimicrobial efficacy of herbal irrigants in endodontic infections. The present study demonstrated that both cryotreated neem extract and cryotreated sodium hypochlorite were effective in reducing post- operative pain, with no significant difference between the groups, suggesting that cryotreated neem may serve as a viable biocompatible alternative. These findings are consistent with previous literature indicating that cold irrigation protocols significantly reduce pain intensity compared to room temperature irrigants [12, 13]. The combined analgesic effect of cryotherapy and neem may be attributed to their complementary mechanisms. Cryotherapy limits tissue injury and reduces inflammatory mediator release, whereas neem inhibits pro-inflammatory enzymes such as cyclooxygenase and lipoxygenase and exhibits strong antimicrobial activity [10]. Within the limitations of this study, these findings provide encouraging evidence for the incorporation of cryotreated herbal irrigants in clinical endodontics to improve patient comfort and reduce post-operative analgesic requirements.

## Conclusion:

We show that both cryotreated neem extract and cryotreated 2.5% sodium hypochlorite significantly reduce post-treatment discomfort after a single-visit root canal procedure in molars exhibiting symptomatic apical periodontitis. Data shows that cryotherapy enhances the analgesic potential of both herbal and conventional irrigants. Cryotreated neem emerges as a promising biocompatible alternative, with comparable effectiveness to sodium hypochlorite and reduced analgesic requirement. Within the limitations of this study, the combination of cryotherapy and neem extract may offer a patient-friendly and effective pain management strategy in endodontics.

## Limitations:

[1] First, the follow-up duration was limited to 7 days; hence, the long-term effects of cryotreated irrigants on periapical healing and pain recurrence could not be evaluated.

[2] Second, the study did not assess inflammatory biomarkers such as interleukins or substance P, which could have provided a deeper understanding of the biological mechanisms behind pain reduction.

[3] Third, although patients were blinded to group assignment, operator blinding during irrigation and instrumentation was not feasible, which could introduce procedural bias.

[4] Finally, the sample was drawn from a single center, limiting the generalizability of results to other populations or clinical settings.

## Future directions:

To strengthen and expand upon the present findings, future research should consider:

[1] Longitudinal studies with extended follow-up periods to evaluate sustained effects on periapical healing.

[2] Biochemical assessments of pro- and anti-inflammatory cytokines (e.g., IL-6, IL-10, substance P) to corroborate pain score trends with molecular evidence.

[3] Multicentric randomized controlled trials with larger sample sizes to enhance the external validity of the results.

[4] Exploring additional herbal extracts with cryotherapy to compare their efficacy against neem and sodium hypochlorite.

[5] Investigating different cryotherapy application methods, such as passive ultrasonic agitation of cold irrigants or extended cold exposure duration.
